# YH29407 with anti-PD-1 ameliorates anti-tumor effects *via* increased T cell functionality and antigen presenting machinery in the tumor microenvironment

**DOI:** 10.3389/fchem.2022.998013

**Published:** 2022-12-05

**Authors:** Dong Kwon Kim, Chun-Bong Synn, Seung Min Yang, Seongsan Kang, Sujeong Baek, Se-Woong Oh, Gyu-Jin Lee, Ho-Woong Kang, Young-Sung Lee, Jong Suk Park, Jae Hwan Kim, Youngseon Byeon, Young Seob Kim, Doo Jae Lee, Hyun-Woo Kim, June Dong Park, Sung Sook Lee, Ji Yun Lee, Jii Bum Lee, Chang Gon Kim, Min Hee Hong, Sun Min Lim, Hey Ryun Kim, Kyoung-Ho Pyo, Byoung Chul Cho

**Affiliations:** ^1^ Severance Biomedical Science Institute, Yonsei University College of Medicine, Seoul, South Korea; ^2^ Brain Korea 21 PLUS Project for Medical Science, Yonsei University College of Medicine, Seoul, South Korea; ^3^ Department of Research Support, Yonsei Biomedical Research Institute, Yonsei University College of Medicine, Seoul, South Korea; ^4^ JEUK Institute for Cancer Research, JEUK Co., Ltd., Gumi, South Korea; ^5^ Yuhan R&D Institute, Yuhan Corporation, Seoul, South Korea; ^6^ Wide River Institute of Immunology, Seoul National University, Hongcheon, South Korea; ^7^ Department of Pediatrics, Seoul National University College of Medicine, Seoul, South Korea; ^8^ Department of Hematology-Oncology, Inje University Haeundae Paik Hospital, Busan, Korea; ^9^ Division of Medical Oncology, Department of Internal Medicine and Yonsei Cancer Center, Severance Hospital, Yonsei University College of Medicine, Seoul, South Korea; ^10^ Yonsei New Il Han Institute for Integrative Lung Cancer Research, Yonsei University College of Medicine, Seoul, South Korea

**Keywords:** IDO1, tryptophan, kynurenine, molecule inhibitor, colon cancer, immunotherapy

## Abstract

Among cancer cells, indoleamine 2, 3-dioxygenase1 (IDO1) activity has been implicated in improving the proliferation and growth of cancer cells and suppressing immune cell activity. IDO1 is also responsible for the catabolism of tryptophan to kynurenine. Depletion of tryptophan and an increase in kynurenine exert important immunosuppressive functions by activating regulatory T cells and suppressing CD8^+^ T and natural killer (NK) cells. In this study, we compared the anti-tumor effects of YH29407, the best-in-class IDO1 inhibitor with improved pharmacodynamics and pharmacokinetics, with first and second-generation IDO1 inhibitors (epacadostat and BMS-986205, respectively). YH29407 treatment alone and anti-PD-1 (aPD-1) combination treatment induced significant tumor suppression compared with competing drugs. In particular, combination treatment showed the best anti-tumor effects, with most tumors reduced and complete responses. Our observations suggest that improved anti-tumor effects were caused by an increase in T cell infiltration and activity after YH29407 treatment. Notably, an immune depletion assay confirmed that YH29407 is closely related to CD8^+^ T cells. RNA-seq results showed that treatment with YH29407 increased the expression of genes involved in T cell function and antigen presentation in tumors expressing ZAP70, LCK, NFATC2, B2M, and MYD88 genes. Our results suggest that an IDO1 inhibitor, YH29407, has enhanced PK/PD compared to previous IDO1 inhibitors by causing a change in the population of CD8^+^ T cells including infiltrating T cells into the tumor. Ultimately, YH29407 overcame the limitations of the competing drugs and displayed potential as an immunotherapy strategy in combination with aPD-1.

## Introduction

In recent decades, immunotherapeutic agents have been used for the treatment of cancer, with immune checkpoint inhibitors (ICIs) representing the most clinically successful strategies (Darvin et al., 2018). Therefore, interest in the development of immuno-oncology drugs has dramatically increased (Meric-Bernstam et al., 2021). However, ICIs employed as a single treatment for cancer retain an average response rate of 10–40% (Hellmann et al., 2016; Sharma et al., 2017; Chen et al., 2021). Moreover, T cells may become exhausted or tumor cells develop drug resistance during treatment (Seliger and Quandt, 2012). Recently, a plethora of clinical trials has tested different treatment combinations to improve the effectiveness of ICIs, with indoleamine 2,3-dioxygenase 1 (IDO1) gaining attention as a candidate for combination therapies (Renner et al., 2017; Marin-Acevedo et al., 2018; Cherney et al., 2021). IDO1 is expressed in vascular cells, macrophages, dendritic cells, and other cells, such as eosinophils, endothelial cells, and fibroblasts (Merlo et al., 2020). Immune checkpoint molecules, such as prostaglandin E2 (PGE2), pathogen-associated molecular pattern, toll-like receptor, damage-associated molecular pattern, interleukin-6 (IL-6), tumor necrosis factor (TNF-α), and transforming growth factor (TGF-β), reportedly upregulate the presence of IDO1 (Spranger et al., 2013; Meireson et al., 2020). IDO1 expression can also be increased through cytokine activity. The crucial role of IDO1 is attributed to the kynurenine (Kyn) pathway of tryptophan (Trp) metabolism, both of which play a role in inhibiting the anti-tumor activation of effector T cells (Amobi et al., 2017).

IDO1 can affect tumor progression in four major ways. First, IDO1 promotes tumorigenesis through the formation of antigen-presenting cells in peripherals that enable immune tolerance of tumor-associated antigens (Tang et al., 2021). Second, IDO1 is an innate immune regulator that converts Trp to Kyn in tumor microenvironments (Yoshida et al., 1979; Munn and Mellor, 2007; Prendergast et al., 2014; Liu et al., 2018). Third, IDO1 induces the activation of regulatory T cells (Tregs) and the suppression of effector T cells, NK cells, and macrophages (Munn et al., 1999; Hwu et al., 2000; Chung et al., 2009). Finally, IDO1 modulates the expression of interferon-γ and interleukin-6, thereby promoting tumor neovascularization (Munn and Mellor, 2013; Liu et al., 2018). Several previous studies have reported that the administration of IDO1 inhibitors improves cancer treatment (Cheong and Sun, 2018; Jung et al., 2019; Lou et al., 2019; Liang et al., 2021; Mo et al., 2021; Odunsi et al., 2022; Zhang et al., 2022). The first-generation IDO1 inhibitor ‘epacadostat,’ which has previously been employed in preclinical studies and confirmed its effectiveness through several clinical studies (stage 1/2), was the subject of a large-scale phase 3 trial, ECHO-301/KN-252. This clinical trial found no benefit in the combined treatment effects of epacadostat and anti-PD-1 (aPD-1) compared with aPD-1 monotherapy in advanced melanoma (Long et al., 2019). The insufficient Kyn inhibitory effect of first-generation IDO1 inhibitors is one of the reasons for the failure of ECHO-301/KN-252 (Van den Eynde et al., 2020; Fujiwara et al., 2022). The second-generation IDO1 inhibitor BMS-986205, administered orally, has been used in 24 clinical studies since its development according to ClinicalTrials.gov. Several stage 1/2 clinical studies have reported that combination treatment with BMS-986205 and nivolumab is safe and can improve the response rate (NCT03192943, NCT02658890). In addition, a clinical study conducted to investigate the combination of BMS-986205 with chemotherapy and *bacillus* Calmette-Guerin demonstrated that BMS-986205 is a better IDO1 inhibitor for treatment than epacadostat (Peng et al., 2020).

In this study, we compare the efficacy of a new drug, YH29407, with that of currently available first- and second-generation IDO1 inhibitors. The anti-tumor effects of YH29407 are determined using both *in vivo* and *in vitro* experiments to evaluate tumor growth inhibition and immune response at the cellular level compared to its competitors. IDO1 is overexpressed in many malignant tumors, but particularly in colorectal cancer (Yu et al., 2022). Furthermore, IDO1 expression is correlated with poor prognosis in clinical outcomes in patients with colorectal cancer (Lou et al., 2019; Mo et al., 2021). Therefore, MC38 colorectal cancer-bearing mice are treated with YH24907, epacadostat, and BMS-986205 to compare and evaluate their anti-tumor effects and changes in immune cell dynamics. According to our results, YH29407, a second-generation novel IDO1 inhibitor, shows a superior anti-tumor effect compared to first-generation epacadostat and second-generation BMS-986205. Interestingly, we observe through immunohistochemistry (IHC), flow cytometry, and *in vitro* assays that T cell machinery is increased in the YH29407+aPD-1 combination group. According to RNA-seq, we also observe increased expression of ZAP70, LCK, NFATC2, and ICAM1, which are genes related to the T cell-mediated pathway, and HLA-DQB1, TAP2, B2M, and MYD88, which are genes related to the antigen-presentation pathway.

## Methods and materials

### Reagents and cell lines

MC38 cells (CVCL_B288) were purchased from Kerafast (Boston, MA, USA). MC38 colorectal tumor cells were cultured in DMEM (ThermoFisher Scientific; Waltham, MA) and supplemented with 10% fetal bovine serum (FBS) (ThermoFisher Scientific) and 1% antibiotic–antimycotic (ThermoFisher Scientific). Cells were grown in a humidified incubator at 37°C with 5% CO2 and tested regularly for *mycoplasma* contamination. Cells were harvested by trypsinization when they reached 70–80% confluence.

The IDO1 inhibitor YH29407 was provided by YUHAN Corp., South Korea. YH29407 was prepared as a 20 mg/ml or 6 mg/ml solution in the formulated buffer (400 ml D.W. + 0.8 ml Tween80 + 2 g Methylcellulose). Epacadostat and BMS-986205, which were purchased from Selleck Chemicals (Houston, TX), were prepared as 20 mg/ml, 20 mg/ml, or 25 mg/ml solutions, respectively, in the above formulated buffer.

### Murine tumor models

Procedures involving the care and use of animals in this study were reviewed and approved by the Institutional Animal Care and Use Committee (IACUC number, 2021-0112) prior to conducting the study. During the study, the care and use of animals were in accordance with the principles outlined in the Guide for the Care and Use of Laboratory Animals, 8th edition, 2010 (National Research Council). For the human SKOV-3 mouse model, seven-week-old BALB/c-nude female mice were purchased and for the MC38 mouse model, six-week-old C57BL/6 female mice were purchased from ORIENT Bio Inc. (Seongnam, South Korea). The mice were then transferred to an animal facility at Yonsei Medical College, where they were established and bred. The animals were monitored for signs of toxicity throughout the treatment period. Tumor volume and body weight were measured four times a week using digital calipers and scales. The body weights of MC38-engrafted C57BL/6 mice were recorded for up to 10 days after treatment to monitor toxicity. Tumor volume was calculated using the equation ‘V = length x width2 × 0.5’ (where ‘length’ = longest diameter and ‘width’ = shortest, perpendicular diameter) (Faustino-Rocha et al., 2013). For *in vivo* xenograft studies, 1 × 106 MC^38^ cells were inoculated subcutaneously into the right flank of female C57BL/6 mice. Measurement of the tumor volume and treatment were initiated individually after the tumor volume reached approximately 50 mm^3^ in all groups.

### Pharmacokinetics and pharmacodynamics experiment

YH29407, BMS-986205 and epacadostat were orally administered to tumor-bearing mice at 100 mg/kg. Blood samples were obtained from jugular vein with lithium heparin capillary tube at 0, 2, 8, 12 and 24 h after administration. Plasma were obtained from blood samples after centrifugation at 13,000 rpm for 2 min. All plasma samples were stored in deep freezer at approximately -80°C before analysis. Tumors were collected from tumor-bearing mice at 24 h after administration. All drugs, tryptophan and kynurenine concentrations in plasma and tumor were determined using liquid chromatography-mass spectrometry.

### *In vivo* cell depletion

Each immune cell subset was depleted by intraperitoneal injection of depletion antibody (400 μg) twice per week, starting a day prior to treatment, as indicated: mouse 2.43 (anti-CD8, BioXCell; Lebanon, NH), mouse GK1.5 (anti-CD4, BioXCell), and mouse PK136 (anti-NK1.1, BioXCell). Macrophages were depleted by intraperitoneal injection of 300 μg of anti-CSF1R (clone AFS98, BioXCell) every alternate day. Mice were injected with 500 μg of anti-BST2 (clone 927, BioXCell) to deplete plasmacytoid dendritic cells (pDC) (Moniz et al., 2010). Control group mice were injected with 400 μg of isotype control (IgG, BioXCell). Depletion of CD8^+^ T cells, CD4^+^ T cells, macrophages, pDC, and NK cells was confirmed by flow cytometry of PBMC (Moynihan et al., 2016).

### Flow cytometry

MC38 tumors were harvested from C57BL/6 mice on day five after single-treatment initiation when the tumor size reached 200 mm^3^. In the case of combination treatment, mice were administered aPD-1 twice for 5 days while treating the drug in the same manner as the single treatment, and the tumors were harvested. Then, the tumors were enzymatically dissociated into single cells with collagenase type I (Worthington Biochemical, Lakewood, NJ, USA) for 1 h at 37°C in a shaker incubator (DAIHAN Scientific; Wonju, KOR), then filtered through a 70-μm cell strainer. Cells were washed with FACS buffer (PBS containing 3% BSA, 0.01% sodium azide, and 1 mM EDTA) and blocked with FcR Blocking Reagent (Miltenyi Biotec; Bergisch Gladbach, GER) at 24°C for 30 min. After fixation, the cells were stained with True-Nuclear™ transcription factor buffer (BioLegend; San Diego, CA, USA) at room temperature for 30 min. The T cell population was stained with anti-CD45 (103110; BioLegend), anti-CD3e (100320; BioLegend), anti-CD4 (100526; BioLegend), anti-CD8 (100750; BioLegend), anti-CD25 (67-0251-82; Invitrogen, Waltham, MA, USA), anti-CD44 (61-0441-82; Invitrogen), anti-FoxP3 (12-5773-82; eBioscience), and anti-CD197 (120108; BioLegend) antibodies. The NK cell population (CD45^+^CD3^−^CD49b+) was stained with anti-CD49b (108906; BioLegend) antibodies. Additionally, we stained the activation markers using anti-ki67 (652413; BioLegend), anti-PD-1 (135218; BioLegend), and anti-interferon-γ (505832; BioLegend) antibodies. Dendritic cell populations (CD45+F4/80-CD11c+MHC I-) were identified using anti-H-2Db (A15443; Invitrogen), anti-CD11c (117343; BioLegend), anti-CD80 (47-4801-82; eBioscience), and anti-CD86 (104729; BioLegend) antibodies. M1 and M2 macrophages were identified using anti-F4/80 (47-4801-82; eBioscience), anti-CD206 (17-2061-80; eBioscience), anti-CD11c, anti-CD80, and anti-CD86. Myeloid derived suppressor cells were identified using anti-CD11b (101216; BioLegend) and anti-Ly-6G (127606; BioLegend). Whole myeloid cells were stained with anti-IDO1 (654004; BioLegend) antibodies. Multicolor flow cytometry analysis was performed using a BD LSR-fortessa™ X-20 instrument (BD Bioscience, Franklin Lakes, NJ, USA). FlowJo software v10 (Tree Star, Ashland, OR, USA) was used for data acquisition and analysis.

### Immunohistochemistry

To quantitatively measure the increase in CD8^+^ T cells and CD3^+^ T cells after YH29407 + aPD-1 treatment, mouse tumor tissues obtained through internal sacrifice were sectioned into five sheets each after formalin-fixed paraffin-embedded preservation and analyzed by IHC. Fluorescence data were re-implemented mixed images of the 3,3′-Diaminobenzidine result through multispectral image analysis, and the CD3^+^ and CD8^+^ cells were stained yellow. All tumor tissue was analyzed, and 15 to 40 fields were analyzed per sample. The results were derived by quantifying the total number of cells and the number of CD3^+^ and CD8^+^ cells per mm2. Whole slide scanning and cell segmentation were performed to quantify the IHC results, and the degree of CD8^+^ and CD3^+^ T lymphocyte infiltration was measured using Vectra Polaris and InForm software. IHC was performed using an automatic staining machine (LEICA BOND RX). Digital images of IHC slides were obtained using a whole slide scanner. Image deconvolution was performed using InForm software. The slides were stained with CD3e (CD3-12, Cell Signaling Technology, Beverly, MA, USA) and CD8α (D4W2Z, Cell Signaling Technology).

### Cell proliferation assay

The mice were sacrificed after 5 days of treatment with IDO1 inhibitors. The spleens were dissected, dissociated into single cells, and counted using a hemocytometer. Cells (2 × 105) were seeded into each well and cultured in RPMI-1640 medium (WelGENE Inc., Daegu, South Korea) containing 10% FBS (ThermoFisher Scientific) and 1% antibiotic–antimycotic solution (ThermoFisher Scientific). To screen the immune cell population of splenocytes and their phenotype, five stimulants consisting of lipopolysaccharide (LPS) (100 ng/ml), concanavalin A (1 μg/ml), gp70-1 (SAPANCSVA), gp70-2 (GQTANATSL, 10 μg/ml), and MC38 cell whole lysate (10 μg/ml) were added to each well and the cells were incubated at 37°C in a humidified atmosphere of 5% CO2 air. After 24 h of exposure to each stimulant, splenocyte proliferation was determined using a Cell Counting Kit-8 (Dojindo Laboratories, Kumamoto, Japan). Cellular proliferation was measured at 450 nm using a microplate reader.

### Cytokine array

Serum was obtained after 5 days of treatment with IDO1 inhibitors and analyzed using a cytokine array. The serum was stored at −80°C until use. Cytokine array analysis was performed according to the manufacturer’s protocol (RayBiotech, Inc., Norcross, GA, USA). Briefly, the membranes were blocked with a blocking buffer for 30 min. The supernatant was incubated with membranes coated with a 96 anti-mouse cytokine antibody cocktail overnight. The membranes were then incubated with biotinylated detection antibodies and streptavidin-peroxidase conjugates overnight. Finally, the membranes were developed, and cytokine proteins were quantified using Image Quant LAS4000 software (Fujifilm; Tokyo, Japan). The expression level of each cytokine was analyzed using ImageJ software (NIH, Bethesda, MD, USA) and the Bioconductor R package for used for assessment.

### RNA sequencing

RNA sequencing data were obtained by requesting tumor FFPE samples from MACROGEN (SEL, South Korea). Total RNA extracted from FFPE samples was processed to prepare the mRNA-sequencing library using the SureSelectXT RNA Direct Library Preparation kit (Illumina, San Diego, California, USA) according to the manufacturer’s instructions. All samples were sequenced on an Illumina sequencer using paired-end 100 bp reads. The raw image data were transformed by base-calling into sequence data and stored in the FastQC (v0.11.7) format. The paired-end reads of the 14 independent samples were trimmed for both PCR and sequencing adapters using Trimmomatic (v0.38; http://www.usadellab.org/cms/?page=trimmomatic). Trimmed reads were aligned using Bowtie2 (v2.3.4.1) and mapped to the reference mouse genome using HISAT2 (v2.1.0). Gene-level read counts were generated using StringTie (v2.1.3b). Significant differential expression was determined using the DESeq2 package (V.1.26) at the gene level. All gene sets in V.7.0 of the Molecular Signatures Database were analyzed by V.4.0.3 of Gene Set Enrichment Analysis (GSEA) and corrected for multiple hypothesis testing. The p-value threshold was set to 0.05. Heatmaps of differentially expressed genes were generated using Prism (V.9.0). To analyze the T cell receptor (TCR) repertoire, High-depth RNA sequencing was performed. TCR sequences were extracted by V.3.0 of MiXCR, and analyzed using TcR packages (V.2.2.4.1).

### Statistical analysis

All data were analyzed for the mean and standard deviation. Statistical analysis of all data from the MC38 mouse model was performed using analysis of variance (ANOVA) with the Mantel-Cox log-rank test of significant differences in GraphPad Prism (version 9.0; GraphPad Software, San Diego, CA) or the Bioconductor R package for assessment. Statistical analysis of flow cytometry data was performed using the Student’s t-test in GraphPad Prism.

## Results

### A novel IDO1 inhibitor, YH29407, outperformed existing first- and second-generation IDO1 inhibitors on Kyn inhibition in tumor tissue and tumor growth inhibition in a MC38 tumor model

**FIGURE 1:**
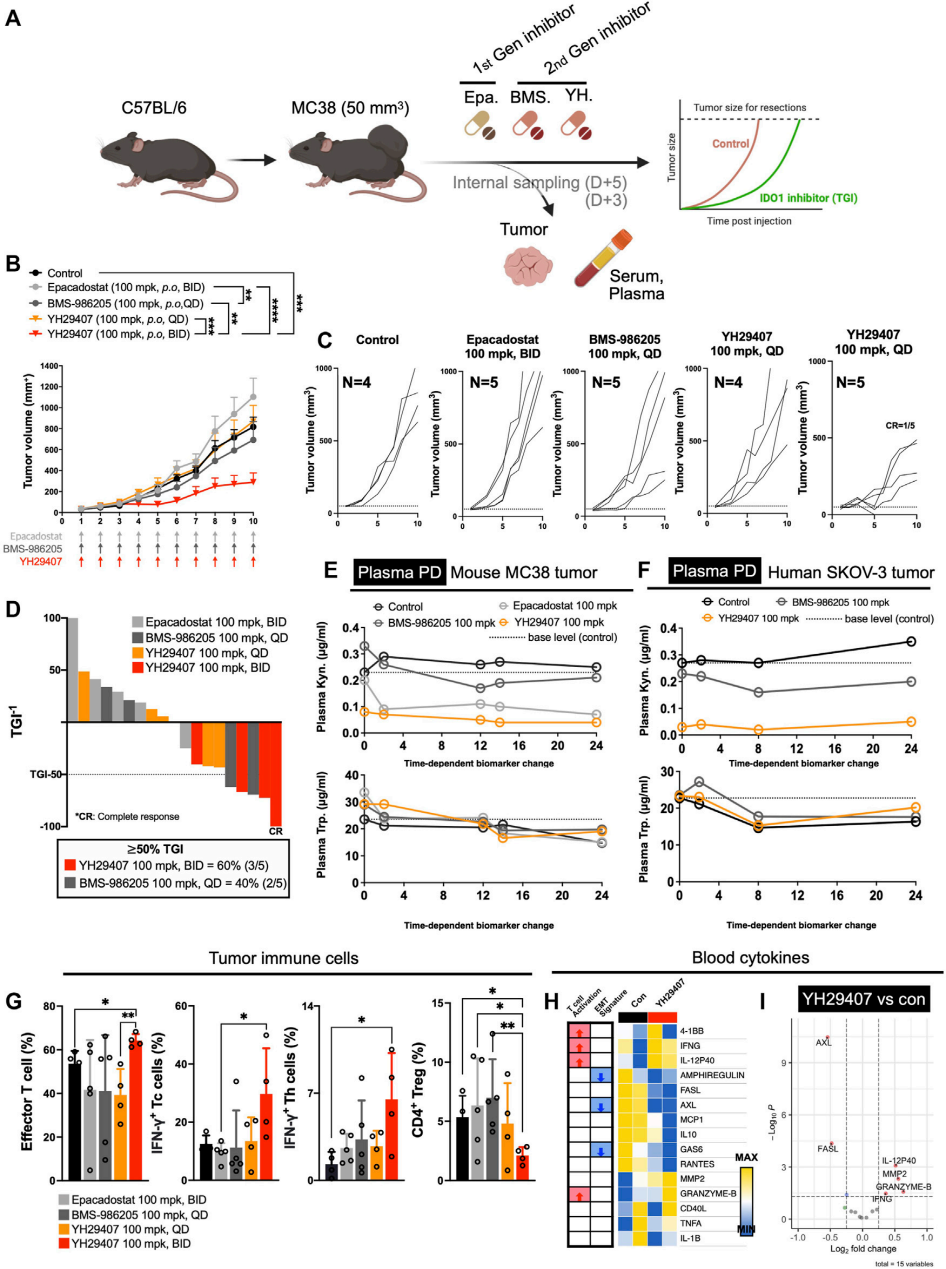
YH29407 with improved pharmacodynamics and pharmacokinetics reduced tumor growth *via* activation of the T cell-mediated immune response in a syngeneic MC38 tumor-bearing model. Syngeneic MC38 colon tumor was grafted onto C57BL/6 mice and treatment was started when the tumor volume reached 50 mm^3^. Epacadostat and BMS-986205 were treated as comparators, and tumor and serum were obtained after 5 days of treatment **(A)**. IDO1 inhibition with YH29407 (100 mg/kg, B.I.D.) significantly suppresses the growth of MC38 tumor in C57BL/6 mice compared to the comparator **(B)**. Tumor volume and number of individual mice **(C)**. Tumor growth inhibition was enhanced by the IDO1 inhibitor, with 60% of mice treated with YH29407 (100 mg/kg, B.I.D.) showing more than 50% TGI **(D)**. Improved pharmacodynamics in the MC38 mouse tumor model **(E)** and human SKOV-3 tumor model **(F)**. **(G)** Analysis of T cell subset by flow cytometry. Effector T cells, IFN-γ+ Tc, and Th cells were increased in the YH29407 (100 mg/kg, B.I.D.) group compared to other groups. However, CD4^+^ Treg was decreased in the YH29407 (100 mg/kg, B.I.D.) group. Heatmap **(H)** and volcano plot **(I)** of cytokine array using serum of YH29407 (100 mg/kg, B.I.D.) and vehicle treated mice showed that EMT signature proteins were decreased and T cell activation cytokines were increased after treatment of YH29407. Statistical significance between groups of **(B)** was calculated using two-way ANOVA followed by Tukey’s multiple comparisons tests (**p ≤ 0.01, ***p ≤ 0.001, ****p ≤ 0.0001), likewise **(G)** was calculated using student’s t-test (*p < 0.05). ANOVA, analysis of variance; Trp, tryptophan; Kyn, kynurenine; IFN-γ+, interferon γ; EMT, epithelial-mesenchymal transition; ANOVA, analysis of variance.

First-generation epacadostat (100 mg/kg, B.I.D.) and second-generation BMS-986205 (100 mg/kg, Q.D.) were subjected to comparative experiments using a dose determined through various reports (Gibney et al., 2019; Sonpavde et al., 2020; Cherney et al., 2021). *In vivo* experiments were designed to confirm the effects of YH29407. MC38 tumors were subcutaneously injected into the right flank of C57BL/6 female mice. Mice were administered the IDO1 inhibitors, i.e., YH29407 (100 mg/kg, Q.D. or B.I.D.), epacadostat (100 mg/kg, B.I.D.), and BMS-986205 (100 mg/kg, Q.D.) when the mean tumor size reached 50 mm^3^ (Figure 1A). To investigate the pharmacokinetics and pharmacodynamics of each drug, all drugs, Trp and Kyn concentrations were determined in the tumor and plasma from MC38 tumor-bearing mice after administration for 3 days (Figure 1E and Supplementary Figure S1A).

YH29407 demonstrated the highest rate of Kyn inhibition in plasma after treatment. (Figure 1E). In contrast, the epacadostat and BMS-986205 treatment groups showed a lower inhibition rate of Kyn. In the plasma of the human SKOV-3 tumor model, YH29407 showed the highest Kyn inhibition rate, with values similar to those shown in Figure 1E (Figure 1F). In addition, YH29407 inhibited Kyn most effectively in tumor tissues (Supplementary Figure S1B). Thus, optimal pharmacodynamics were observed in the YH29407 treatment group. In addition, the pharmacokinetics were evaluated using the MC38 tumor model (Supplementary Figure S1A). Improved pharmacokinetics were also demonstrated in the YH29407 treatment group compared to the BMS-986205 treatment group in plasma and tumor tissue. Compared to the epacadostat-treated group, pharmacokinetics were improved in plasma and similar in tumor tissue.

After confirming the pharmacokinetics/pharmacodynamics of YH29407, the anti-tumor effects of IDO1 inhibitors were evaluated. MC38 tumor-bearing mice were treated with YH29407, epacadostat, and BMS-986205 (Q.D. or B.I.D.) until the tumor volume in the control group reached 1,200 mm^3^ (Figure 1B). The YH29407 (B.I.D.) group showed the best anti-tumor effects on tumors. A significant reduction in tumor growth was primarily observed at day six between the epacadostat and YH29407 (B.I.D.) groups (*p < 0.05). On day eight, tumor volume was significantly reduced in the YH29407 (B.I.D.) group compared with the epacadostat, vehicle, and YH29407 (Q.D.) groups (****p ≤ 0.0001, *p < 0.05, respectively, Figure 1B). On day 10, the tumor size of the YH29407 (B.I.D.) group showed a significantly reduced volume compared to the epacadostat (****p ≤ 0.0001) and BMS-986205 groups (**p ≤ 0.01). In addition, the tumor growth inhibition (TGI) graph showed that groups with TGI >50% were predominantly YH29407 (B.I.D., 60%, Figure 1D). According to the individual tumor size graph within each group, the YH29407 (B.I.D.) group showed the most consistent decrease, with one complete response among the five mice (Figure 1C). In summary, tumor reduction was observed after treatment with IDO1 inhibitors compared to the control group, where the novel YH29407 drug demonstrated the greatest anti-tumor effects.

To determine the immunological response to IDO1 inhibitors, tumor tissue obtained from internal sacrifice on day five was analyzed using flow cytometry to observe changes in immune cell populations after drug treatment (Figure 1G). To examine tumor-infiltrating lymphocytes within the TME, we employed the gating strategy to analyze the ratio of various immune cell subsets, as shown in Supplementary Figure S3. The effector T cells and IFN-γ+ Th cells were significantly increased in the YH29407 (B.I.D.) group compared to those in the control group (*p < 0.05, Figure 1G). The YH29407 (B.I.D.) group showed an increase in IFN-γ+ cytotoxic T cells compared with the epacadostat group (*p < 0.05). It was also confirmed that CD4^+^ regulatory T cells were reduced in the YH29407 (B.I.D.) group compared to those in the control and epacadostat groups (*p < 0.05). Then, the activation of T cells and cytokine profiles were compared between the control and YH29407 groups using serum samples (Figure 1A) by luminex-based cytokine array (Figure 1H and Figure 1I). The most notable increases in the profile were granzyme B and IFN-γ (markers of T cell activation) in the YH29407 (B.I.D.) group. AXL and FASL proteins related to EMT signatures were reduced in the YH29407 (B.I.D.) group compared to those in the control group (Figure 1H and Figure 1I). Based on these findings, the administration of YH29407 twice a day led to more efficient activation of T cells and greater anti-tumor effects in comparison to the administration of currently available first- and second-generation IDO1 inhibitors.

### YH29407 stimulated the proliferation of tumor-specific immune cell populations

**FIGURE 2:**
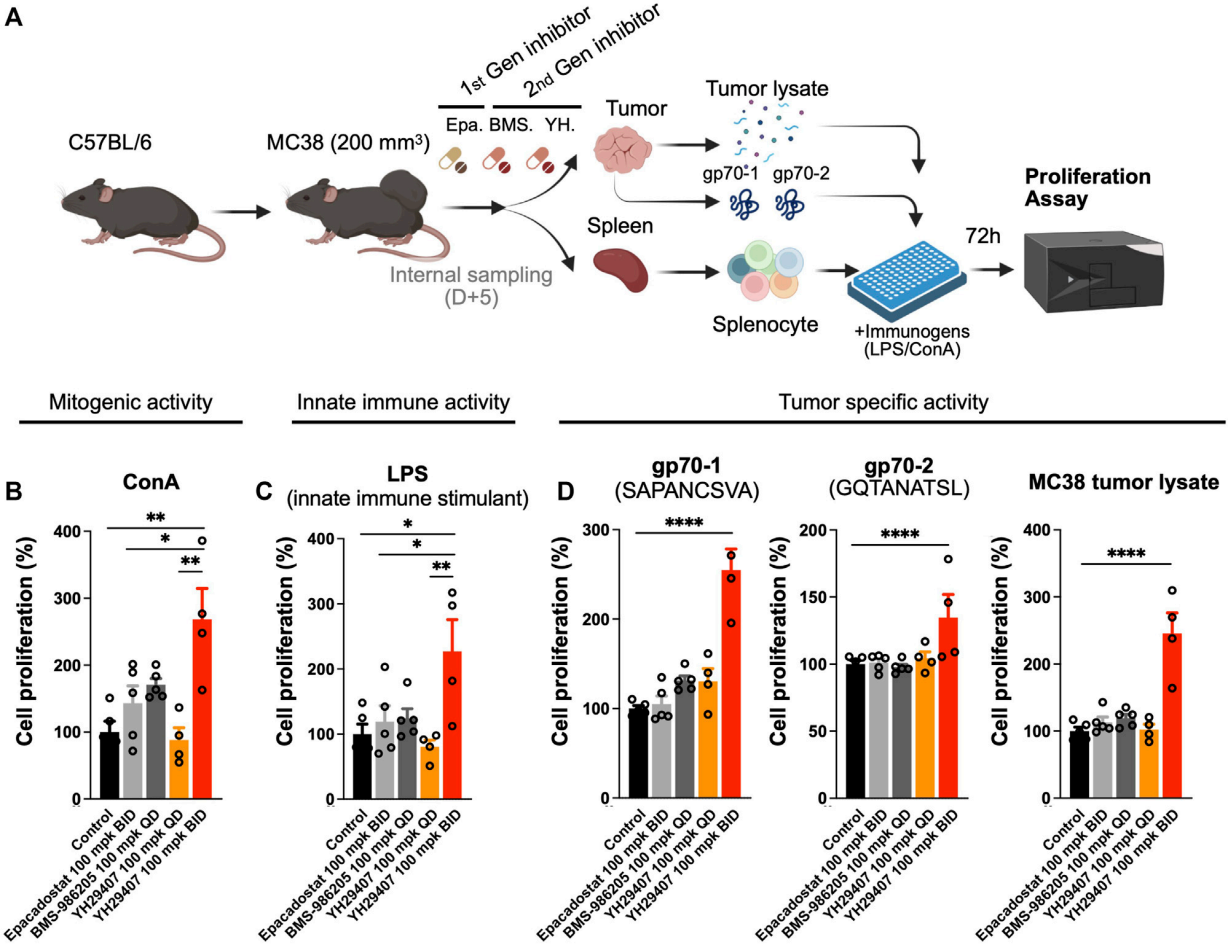
Proliferation of immune cells treated with YH29407 was significantly increased after stimulation with tumor-associated antigen (gp70-1, gp70-2). **(A)** MC38 tumor was grafted onto C57BL/6 mice and 5 days of treatment was started after the tumor volume reached 200 mm^3^. Then, splenocytes were obtained and cells were seeded after being counted using a hematocytometer, and the cells were stimulated with MC38 tumor lysate, gp70-1, gp70-2, ConA, and LPS for 72 h. Cell proliferation assay showed T cell **(B)** and innate immune activation **(C)**. Moreover, tumor-specific T cells were enriched after stimulation with MC38 tumor lysate and tumor-associated antigen **(D)**. *p < 0.05, **p ≤ 0.01, ****p ≤ 0.0001. ConA, concanavalin A; LPS, lipopolysaccharide; IFN-γ+, interferon gamma.

The cytokine assay confirmed that YH29407 B.I.D. treatment induced cytokine release, which promoted changes in the immune cell population against the tumor. To determine the stimulatory effect of the novel IDO1 inhibitor on the T cell population, YH29407-administered mice were examined for immune cells associated with tumor antigens. For this analysis, spleen samples obtained through internal sacrifice after 5 days of treatment were used to analyze systemic immune cell changes. Concanavalin A (ConA), LPS or MC38 tumor lysate, gp70-1, and gp70-2 were used to extract splenocytes to test for T cell mitogenic activity, innate immune stimulation, or tumor-specific activity for 72 h, respectively, to compare changes in systemic immune responses (Figure 2A). When treated with ConA, cell proliferation through mitogenic activity in the YH29407 (B.I.D.) group significantly increased compared to that in the other groups (*p < 0.05, **p ≤ 0.01, Figure 2B). Immune cells from the YH29407 (B.I.D.) group also demonstrated significantly increased sensitivity to innate immune stimulants by LPS compared with the control and other treatment groups (Figure 2C). The increased proliferative responses of the B.I.D YH29407 group (to mitogenic and innate immune signals) were coupled with a significant tumor-specific proliferation of cells in response to gp70-1, gp70-2, and MC38 tumor lysates (****p ≤ 0.0001, Figure 2D). These findings suggest that YH29407 could have synergistic effects with aPD-1 in combination immunotherapy by minimizing T cell exhaustion and optimizing T cell expansion.

### Combination therapy of YH29407 and aPD-1 enhanced tumor growth inhibition and survival rate in comparison to a single treatment

**FIGURE 3:**
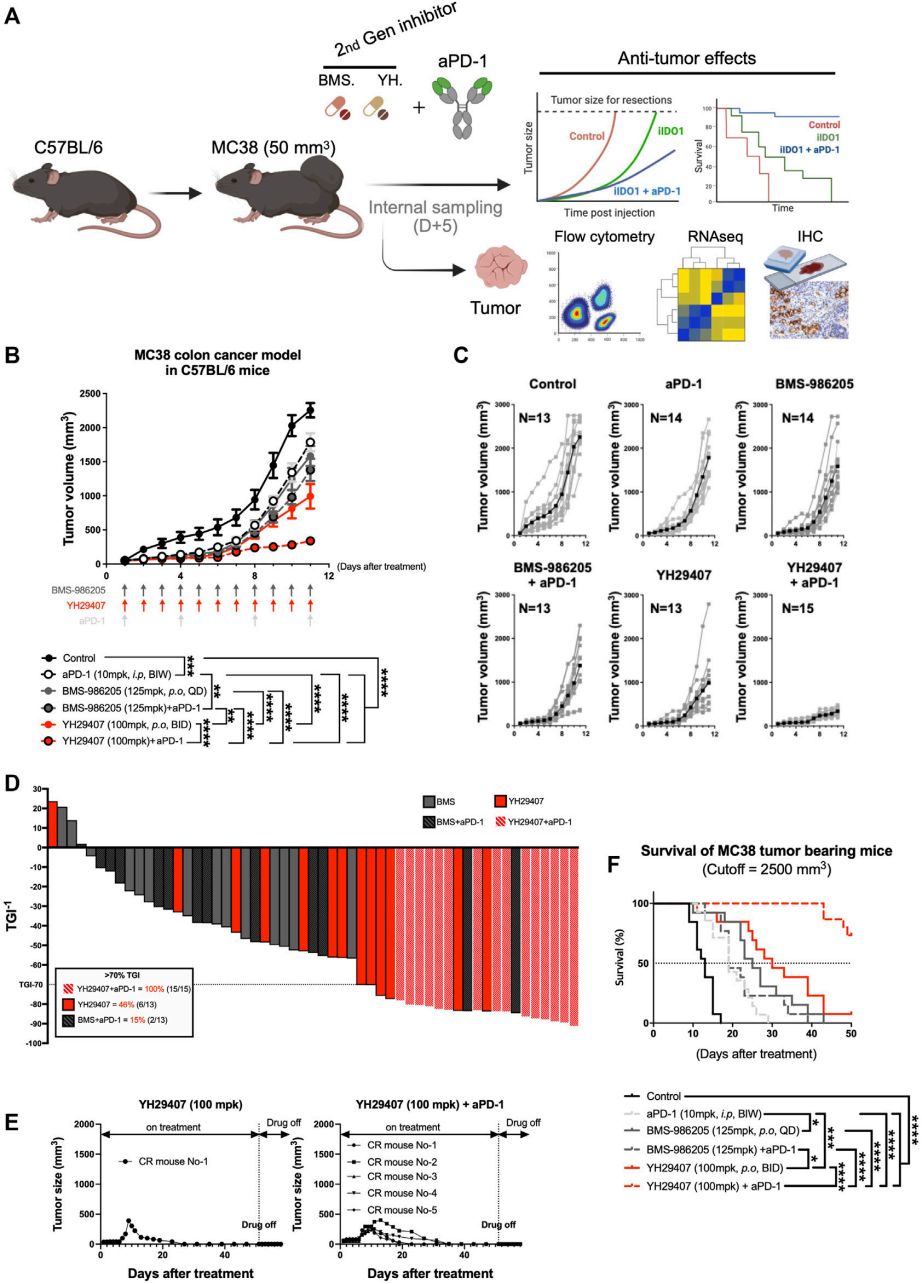
Combination treatment of YH29407 and aPD-1 delayed tumor growth and enhanced tumor growth inhibition and survival of syngeneic MC38 tumor-bearing model. Syngeneic MC38 colon tumor was grafted onto C57BL/6 mice and treatment of YH29407 with or without aPD-1 was started when the tumor volume reached 50 mm^3^. BMS-986205 was treated as comparative drugs, and the control group was treated with a vehicle. **(A)** Schematic design of experiment. **(B)** Combination treatment with YH29407 and aPD-1 significantly delayed MC38 tumor growth in C57BL/6 mice compared to treatment with BMS-986205 or aPD-1 alone. **(C)** Tumor volume and number of individual mice. **(D)** Tumor growth inhibition was enhanced by IDO1 inhibitor; all mice treated with YH29407+aPD-1 showed more than 70% TGI. **(E)** Complete response was observed in the treatment group of YH29407 alone and YH29407 + aPD-1. **(F)** YH29407+aPD-1 treatment group significantly enhanced the overall survival of the MC38 engrafted C57BL/6 mice model. Statistical significance between groups was calculated using two-way ANOVA followed by Tukey’s multiple comparisons tests (*p < 0.05, **p ≤ 0.01, ***p ≤ 0.001, ****p ≤ 0.0001). aPD-1, anti-PD-1 antibody; ANOVA, analysis of variance.

As YH29407 monotherapy increased T cell priming and reactivity, we hypothesized that combination therapy with aPD-1 has synergistic anti-tumor effects so performed a combination test. MC38 tumors were subcutaneously injected into the right flank of C57BL/6 female mice. The anti-tumor effects of YH29407 (100 mg/kg, B.I.D.) with and without aPD-1 (10 mg/kg, B.I.W.) were compared against BMS-986205 (125 mg/kg, Q.D.) with and without aPD-1 throughout tumor progression to evaluate the combinatorial effect (until the tumor volume of the control group reached approximately 2,000 mm^3^, Figure 3A). The most efficient anti-tumor effects were observed in the group treated with a combination of YH29407 and aPD-1 (Figure 3B). The combination treatment of YH29407 and aPD-1 resulted in significant tumor reduction compared to the control as early as day four (*p < 0.05, Figure 3B) and effectively inhibited tumor growth by the endpoint (100% > 70% TGI in 15 mice, Figure 3D). In contrast, a single treatment with YH29407 showed significant tumor reduction from the eighth day compared to the control group and resulted in a comparatively lower frequency of >70% TGI at the endpoint (46% mice, Figure 3D). The anti-tumor effects of YH29407 were evidently enhanced by the addition of aPD-1 in combination therapy. This combination also demonstrated superior TGI compared with BMS-986205 with and without aPD-1 treatment (****p ≤ 0.0001, Figure 3B). Bodyweight was observed to monitor the toxicity of IDO1 inhibitors and aPD-1, and no abnormal changes were observed (Supplementary Figures S2A,B).

A clear difference was also seen in the tumor size graph of individual mice (Figure 3C); five complete responses were observed in the YH29407 + aPD-1 combination treatment group, whereas only one complete response was observed in the YH29407 single-treatment group. Interestingly, the reduction in tumor growth was maintained post-treatment (Figure 3E). In addition, all mice in the YH29407 + aPD-1 combination treatment group were included in the TGI>70% group, unlike the other groups, confirming the synergistic effects on tumor suppression (Figure 3D). The survival rate of the mice was also evaluated to determine whether the tumor suppressive effect of the inhibitors affected survival after treatment (Figure 3F). The endpoint of survival was established when the tumor volume reached 2,500 mm^3^. Mice in the YH29407 + aPD-1 combination treatment group showed a significant increase in survival compared to the other groups (****p ≤ 0.0001). In conclusion, YH29407 exerted comparatively stronger anti-tumor effects on tumor growth and a higher survival rate in an MC38 syngeneic model; however, this effect was significantly enhanced when treated in combination with aPD-1.

### YH29407 + aPD-1 treatment enhanced the activation of cytotoxic CD8^+^ T cells of the immune system in syngeneic models

**FIGURE 4:**
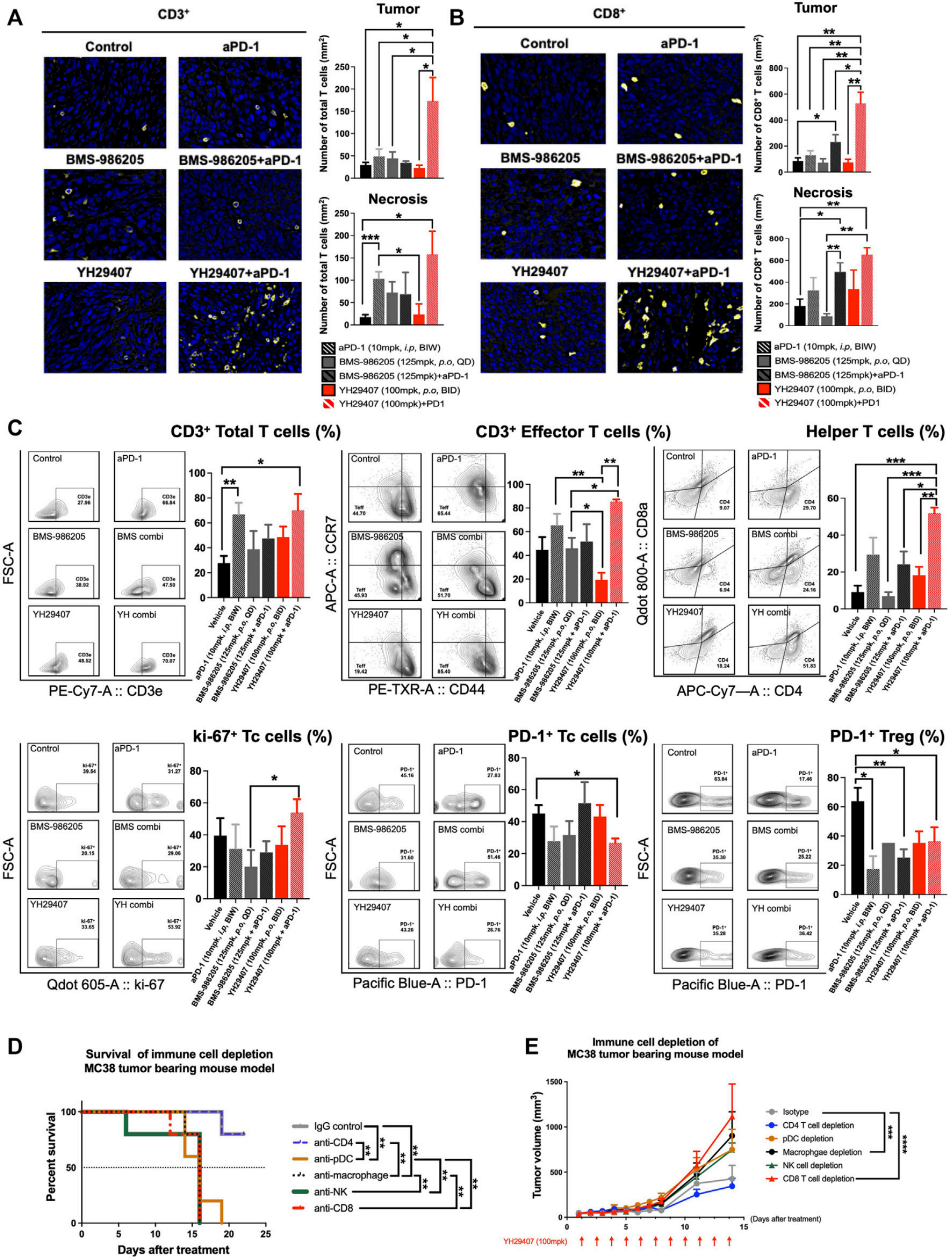
Combination treatment of YH29407 and aPD-1 enhanced T cell activation, particularly effector T cells and helper T cells. Infiltrated CD3^+^ T cells **(A)** and CD8^+^ T cells **(B)** to the tumor were significantly enhanced in the YH29407+aPD-1 group compared to the other groups. **(C)** Total T cells, effector T cells, helper T cells, and ki-67 + Tc cells were significantly increased in the YH29407+aPD-1 group. However, PD-1+ Tc cells and Treg were decreased in the YH29407+aPD-1 group compared to the control and other groups. Immune cell depletion affected survival **(D)** and tumor growth in MC38 tumor-bearing mice **(E)**. Statistical significance between groups was calculated using two-way ANOVA followed by Tukey’s multiple comparisons test or student’s t-test (*p < 0.05, **p ≤ 0.01, ***p ≤ 0.001, ****p ≤ 0.0001). Tc, cytotoxic T cell; Treg, regulatory T cell; ANOVA, analysis of variance.

To investigate changes in the immune population and infiltrating subset after YH29407+aPD-1 combination treatment, tumor tissues obtained through internal sacrifice were subjected to FFPE. Each FFPE sample was processed into five sections, which were analyzed using IHC. CD3^+^ and CD8^+^ cells are stained yellow in Figure 4A and Figure 4B, respectively. There was a dramatic increase in the population of CD3^+^ and CD8^+^ T cells in the YH29407 and aPD-1 combination treatment group compared to that in the other groups. The number of CD3^+^ T cells within the tumor from the YH29407 and aPD-1 combination treatment group was significantly higher than that in the control and all other groups (*p < 0.05, Figure 4A). Additionally, the same trend was observed in the population of CD8^+^ T cells within the tumor and the infiltrating T cells, where the T cells in the YH29407 and aPD-1 combination treatment group were higher than those in the control and all other groups (Figure 4B).

Thus, YH29407 and aPD-1 combination treatment enhanced the infiltration of CD3^+^ and CD8^+^ T cells. Tumor cells obtained through internal sacrifice were analyzed to investigate T cell subpopulations residing in the TME (Figure 4C). The effector T cells were significantly increased in the YH29407 and aPD-1 combination treatment group compared to single BMS-986205 and YH29407 treatment groups (*p < 0.05, **p ≤ 0.01, respectively). Interestingly, proliferating cytotoxic T cells (ki-67+) were significantly increased in the YH29407 and aPD-1 combination group compared to the BMS-986205 single-treatment group (*p < 0.05). The largest difference was observed in the number of helper T cells. Helper T cells were significantly increased in the YH29407 and aPD-1 combination group compared to the control (***p ≤ 0.001), BMS-986205 with (*p < 0.05) and without (***p ≤ 0.001) aPD-1, and single YH29407 (**p ≤ 0.01, Figure 4C) groups. In contrast, the expression of PD-1 in Treg and cytotoxic T cells significantly decreased in the YH29407+aPD-1 group compared to that in the control group (*p < 0.05, Figure 4C).

To confirm whether changes in immune cells influenced the anti-tumor effects, immune cell depletion experiments were performed *in vivo*. The most rapid tumor growth occurred upon depletion of CD8^+^ T cells (****p ≤ 0.0001, Figure 4E). There were no significant tumor suppressive effects by depletion of NK cells and pDCs (immune cells possibly affected by IDO) compared to the control; however, depletion of macrophages significantly affected tumor growth (***p ≤ 0.001, Figure 4E). Survival rate analysis was performed based on a tumor volume of 1,000 mm^3^; interestingly, only depletion of CD4 mimicked the survival rate of the control (**p ≤ 0.01, Figure 4D).

### T cell-mediated tumor suppression was reflected in gene expression and related pathways

**FIGURE 5:**
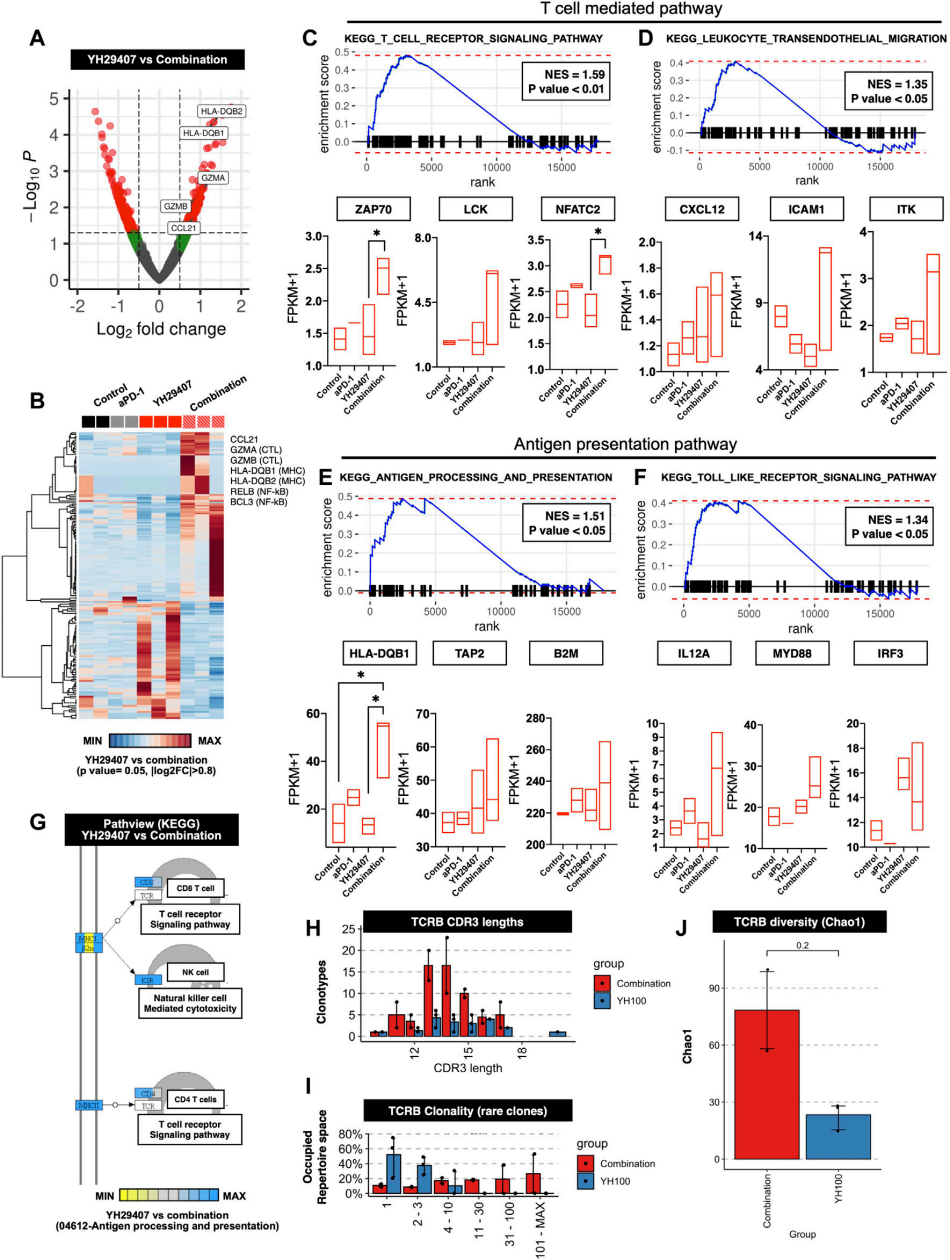
Differentially expressed genes (DEGs) and gene set enrichment analysis (GSEA) between the progression of YH29407+aPD-1 and other treatment groups. **(A)** Volcano plots of 17,871 genes used to compare DEGs in YH29407+aPD-1 and YH29407 treatment. DESeq2 package was used for folds of gene expression and statistical analysis (folds ≧|2|, *p* < 0.05). Individual gene expression and p values are displayed as colored dots (gray = non-significant and non-fold changes, green = folds >|2|, red = folds ≧|2|, and *p* < 0.05). Compared to YH29407 alone, lower expression of 138 genes and higher expression of 221 genes were identified in the YH29407+aPD-1 group. **(B)** Heatmaps show the core enrichment genes of each group. Gradient key color presents the expression of individual genes (Z-score normalized). **(C–F)** GSEA and KEGG pathway. Among the significant gene sets, T cell receptor signaling pathway, leukocyte transendothelial migration, antigen processing, and presentation and toll-like receptor signaling pathways are demonstrated with their normalized enrichment score (NES) and p value. **(G)** Pathview of KEGG pathway. **(H)** CDR3 length of TCR-beta analyzed by MiXCR and TcR analysis packages. **(I)** Rare clonality was expanded in the combination group. **(J)** TCRB diversity was increased in the combination group.

Figures 1–4 shows that YH29407 increased the T cell-mediated anti-tumor effects; therefore, we expected that the T cell machinery-related transcriptome would also increase. To expand our understanding of the immune phenotype, RNA sequencing analysis of the YH29407 single treatment and YH29407 and aPD-1 combination treatment groups was performed. Comparisons of volcano plots between the single and combination treatment groups identified 359 transcripts (221 genes upregulated, 138 genes downregulated) that were differentially expressed (Figure 5A). For this analysis, cytokines and transcription factors were analyzed, as shown in the heat map (Figure 5B). It demonstrated specific gene clusters located in each group, with the YH29407 and aPD-1 combination group showing upregulated genes associated with T cell signaling and cytolytic activity. In particular, T cell-mediated and antigen-presentation pathways were likely involved in tumor suppression according to GSEA analysis (Figures 5C–F). GSEA also revealed that the TCR signaling pathway (GO), antigen processing, and presentation (GO) were significantly enriched in the YH29407 and aPD-1 combination treatment group (Figures 5C,E,G). Leukocyte transendothelial migration (GO) and toll-like receptor signaling pathways were also increased in the YH29407 and aPD-1 combination group; however, the differences were not significant (Figure 5D and Figure 5F). Combination treatment with YH29407 and aPD-1 significantly increased the expression of NFATC2 and HLA-DQB1 compared to that in the single YH29407 treatment group (*p < 0.05, Figure 5C and Figure 5E). NFATC2 and HLA-DQB1 played key roles in suppressing tumor growth by increasing T cell machinery through the activation of T cells and antigen presentation, respectively. These data agree with the data in Figures 1–4, which indicate that CD8^+^ and CD4^+^ T cells increased after YH29407 and aPD-1 combination treatment. To identify clonally expanded cells as indicators of tumor specificity, we analyzed the TCR sequence. Compared to YH29407 alone, YH29407+aPD-1 combination treatment showed a change in CDR3 length, which could affect TCRB (which encodes TCRβ). Moreover, it was confirmed that the clonality of the rare clone was expanded in the combination treatment group (Figure 5H and Figure 5I). In addition, the diversity score was higher in the combination group; however, the difference was not statistically significant (Figure 5J).

## Discussion

Since its discovery, IDO has contributed to important research outcomes and achievements (Sharma et al., 2021; Zhai et al., 2021; Zhang et al., 2021; Nandre et al., 2022). It is well known that the IDO enzyme, which converts tryptophan, an essential amino acid, into Kyn, inhibits cells such as CD8^+^ T cells, NK cells, and macrophages, activates regulatory T cells, and has positive effects on tumor progression (Mbongue et al., 2015; Zhai et al., 2020). Therefore, several clinical trials have used IDO inhibitors to induce immune activation and tumor suppression by inhibiting IDO (NCT04106414, NCT01685255, and NCT03695250). Furthermore, various pharmaceutical companies have participated in the development of IDO1 inhibitors. In particular, epacadostat, a first-generation IDO1 inhibitor, has achieved success in entering phase 3 clinical trials. However, it has not yet passed phase 3 clinical trials (NCT02752074) for reasons already identified, including 1) the dose was not clearly set up, 2) the pharmacokinetics/pharmacodynamics were not sufficiently maintained, and 3) IDO2 and TDO enzymes should be inhibited as well as IDO1.

A second-generation IDO1 inhibitor, ‘BMS-986205′, which surpasses the first-generation epacadostat, has also been developed. To date, two active clinical trials and various studies using BMS-986205 in combination with aPD-1 are in progress. In comparing with BMS-986205, we suggest the best-in-class ‘YH29407.’ YH29407 is a novel IDO1 inhibitor that has high pharmacokinetics and pharmacodynamics compared to previously developed IDO1 inhibitors, and shows synergy in combination treatment with aPD-1. First, we tested a single treatment with YH29407 and its competitors. In a pharmacodynamic study, YH29407 suppressed Kyn most effectively in tumor tissues, and it was shown to have improved pharmacodynamics compared to existing IDO1 inhibitors. In addition, the pharmacokinetic study revealed that the drug concentration in the tumor remained highest for the longest time. However, despite the high pharmacokinetic levels in the plasma, our data showed that YH29407 itself was well tolerated without observable abnormalities such as body weight or behavioral changes in mice treated with YH29407. Previously, several studies have reported the metabolism that converts Trp to Kyn by the IDO enzyme. Moreover, various IDO1 inhibitors have been shown to inhibit Trp metabolism, which promotes tumor progression.

Compared to BMS-986205 and epacadostat, the YH29407 (100 mg/kg, B.I.D.) single-treatment group showed the best anti-tumor effects. As expected, the results showed a synergistic effect when YH29407 was combined with aPD-1. A significant increase in survival and inhibition of tumor growth was confirmed in the group administered YH29407 + aPD-1 compared to the group YH29407 administered alone. In particular, five complete responses were observed in the YH29407 + aPD-1 combination group.

Our observations using YH29407 showed that many types of immune cells were associated with Trp metabolism by the IDO1 enzyme. In particular, the immune depletion assay indicated that CD8^+^ T cells, NK cells, macrophages, and pDCs were associated with tumor growth and survival. However, a similar trend was not observed for CD4^+^ T cells. We propose that the depletion of CD4^+^ T cells is not associated with tumor growth and survival, as the increase in CD4^+^ regulatory T cells by Kyn and aryl hydrocarbon receptors from Trp metabolism is primarily used to suppress other immune cells that have killing effects. Rather, because immune cells with killing effects were activated by YH29407, which is an IDO1 inhibitor with high pharmacokinetics and pharmacodynamics, the inhibition of CD4^+^ T cells, which can differentiate into Tregs, may have helped in tumor suppression.

Most importantly, our overall data converged to the conclusion that activating T cell immunity was the cause of tumor suppression. In the YH29407+aPD-1 treatment group, the HLA- type gene and cytotoxicity-related genes, granzyme A and B, were increased compared to YH29407 treatment alone. In addition, it was observed that the T cell-mediated and antigen-presentation pathways were increased in the YH29407+aPD-1 treatment group compared to the control, aPD-1 alone, and YH29407 alone groups.

In conclusion, we analyzed the effects of IDO1 inhibitors on tumor suppression, and provide new insights into their association with immune cells, especially CD8^+^ T cells. Even though we conducted other immune cell test including CD4^+^, Macrophage, Dendritic cells there were no significant affection in tumor regression. We analyzed with RNA sequencing and evaluated with immune depletion assay T cell-mediated tumor suppression and found that it was directly related to immune cell depletion. Therefore, our results show that YH29407, which has improved pharmacokinetics and pharmacodynamics, is the best-in-class IDO1 inhibitor that suppresses tumors and exhibits synergistic effects with immuno-oncology agents such as aPD-1 through the effective blocking of IDO1 enzymes. Since aPD1 + YH29407 combination strategy showed the best anti-tumor effects than others, our findings suggest that an effective block of the IDO1 enzyme may form an ideal combination with an immune checkpoint inhibitor for cancer treatment.

## Data Availability

The data presented in the study are deposited in the Figshare repository. The data can be downloaded at the following repository website: https://figshare.com/articles/dataset/YH29407_20221103_/21482955.
