# Supplementary figures and images for: YH29407 with anti-PD-1 ameliorates anti-tumor effects via increased T cell functionality and antigen presenting machinery in the tumor microenvironment
Source: Front Chem. 2022 Dec 5;10:998013. doi: 10.3389/fchem.2022.998013 (PMC9761775; doi:10.3389/fchem.2022.998013)

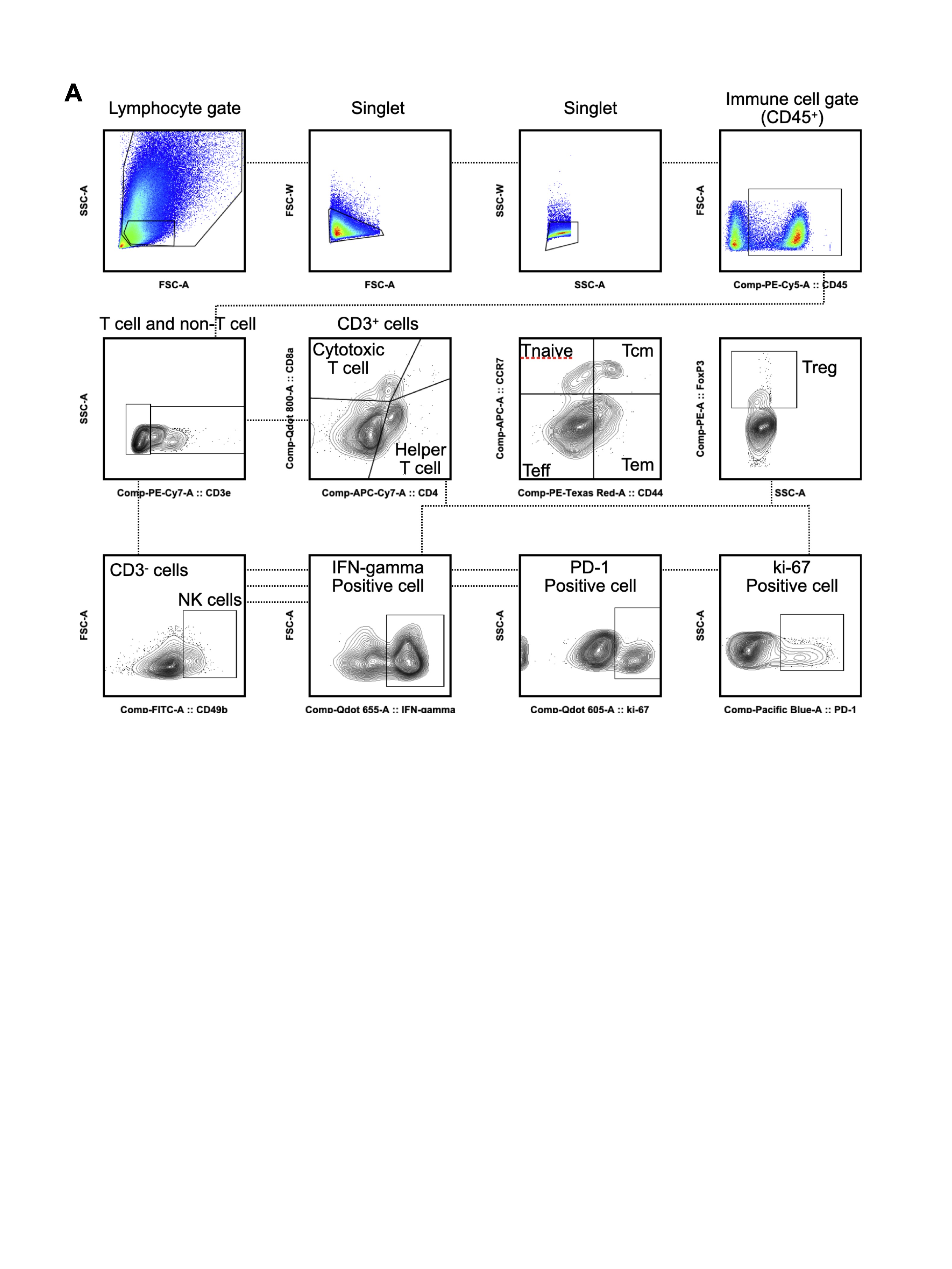

Supplement: Supplementary file 1 [file Image3.JPEG]

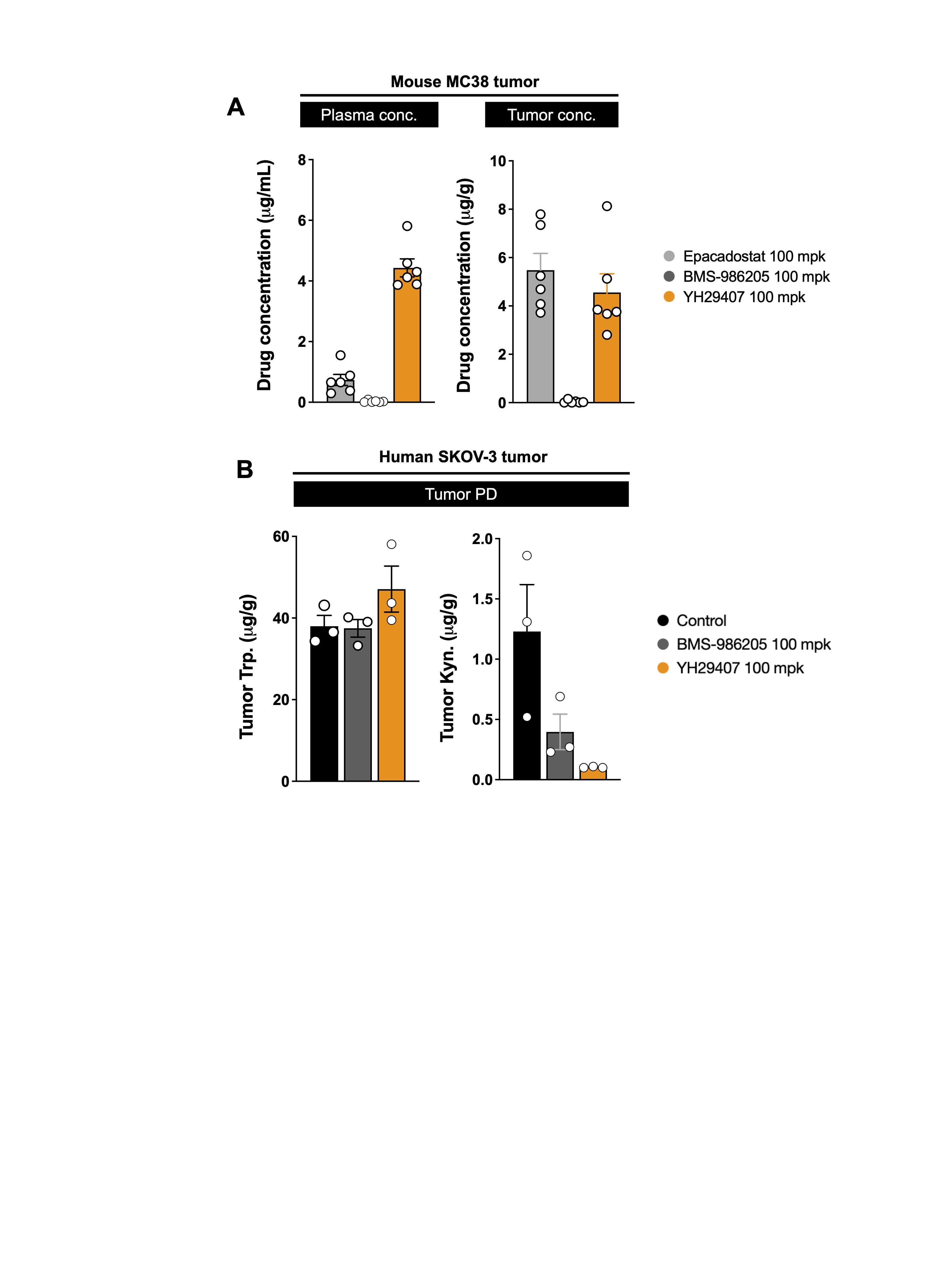

Supplement: Supplementary file 2 [file Image1.JPEG]

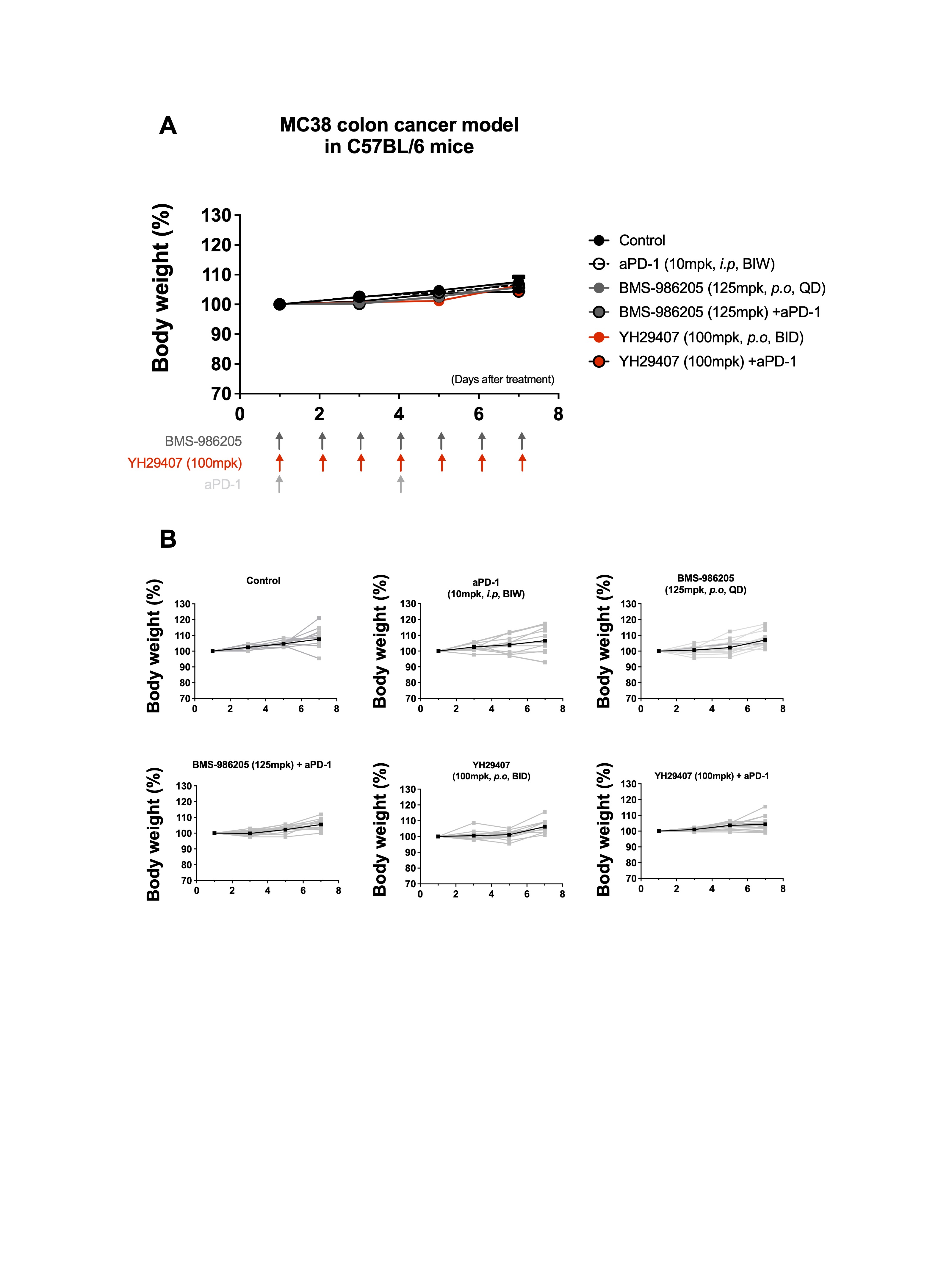

Supplement: Supplementary file 3 [file Image2.JPEG]
