# Supplementary material for: YH29407 with anti-PD-1 ameliorates anti-tumor effects via increased T cell functionality and antigen presenting machinery in the tumor microenvironment
Source: Front Chem. 2022 Dec 5;10:998013. doi: 10.3389/fchem.2022.998013 (PMC9761775; doi:10.3389/fchem.2022.998013)
Supplement: Supplementary file 4 [file DataSheet1.docx]

**Supplementary Figure 1** | YH29407 showed improved pharmacodynamics and pharmacokinetics in the mouse model. The novel IDO1 inhibitor "YH29407" showed improved pharmacokinetics in the MC38 tumor-bearing mouse model (**A**). The greatest inhibition of Trp-to-Kyn conversion was observed in the YH29407 treatment group in the human SKOV-3 tumor model. Trp, tryptophan; Kyn, kynurenine.

**Supplementary Figure 2** | Body weight of each group. Average body weight and SEM of animals measured on different days. Body weight change of (**A**) group average and (**B**) individual mice. Body weight of Day 1 = 100%. To evaluate side effects such as YH29407 toxicity, body weight was measured four times for up to seven days. When the first day was set to 100%, the average body weight for each group was maintained at 100% or more. Lower results show the weight of each individual mouse.

**Supplementary Figure 3** | Gating methods for analysis of T cell subsets in tumors.
